# Diagnostic yield of exome sequencing in myopathies: Experience of a Slovenian tertiary centre

**DOI:** 10.1371/journal.pone.0252953

**Published:** 2021-06-09

**Authors:** Ivana Babić Božović, Aleš Maver, Lea Leonardis, Marija Meznaric, Damjan Osredkar, Borut Peterlin

**Affiliations:** 1 Clinical Institute of Genomic Medicine, University Medical Centre Ljubljana, Ljubljana, Slovenia; 2 Institute of Clinical Neurophysiology, University Medical Centre Ljubljana, Ljubljana, Slovenia; 3 Department of Neurology, Faculty of Medicine, University of Ljubljana, Ljubljana, Slovenia; 4 Institute of Anatomy, Faculty of Medicine, University of Ljubljana, Ljubljana, Slovenia; 5 Department of Paediatric Neurology, University Medical Centre Ljubljana, Ljubljana, Slovenia; Oslo Universitetssykehus, NORWAY

## Abstract

**Background:**

Our aim was to present the experience of systematic, routine use of next generation sequencing (NGS) in clinical diagnostics of myopathies.

**Methods:**

Exome sequencing was performed on patients with high risk for inherited myopathy, which were selected based on the history of the disease, family history, clinical presentation, and diagnostic workup. Exome target capture was performed, followed by sequencing on HiSeq 2500 or MiSeq platforms. Data analysis was performed using internally developed bioinformatic pipeline.

**Results:**

The study comprised 86 patients, including 22 paediatric cases (26%). The largest group were patients referred with an unspecified myopathy (47%), due to non-specific or incomplete clinical and laboratory findings, followed by congenital myopathies (22%) and muscular dystrophies (22%), congenital myotonias (6%), and mitochondrial myopathies (3%). Altogether, a diagnostic yield was 52%; a high diagnostic rate was present in paediatric patients (64%), while in patients with unspecified myopathies the rate was 35%. We found 51 pathogenic/likely pathogenic variants in 23 genes and two pathogenic copy number variations.

**Conclusion:**

Our results provide evidence that phenotype driven exome analysis diagnostic approach facilitates the diagnostic rate of complex, heterogeneous disorders, such as myopathies, particularly in paediatric patients and patients with unspecified myopathies.

## Introduction

Myopathies represent a clinically and genetically heterogeneous group of neuromuscular disorders that commonly represent a diagnostic challenge [1,2]. They are characterized by a highly variable, sometimes non-specific clinical presentation and frequently overlapping phenotypes between different diseases. Mutations over 200 genes have been implicated in myopathies, including some of the largest genes in humans [2]. The genotype-phenotype correlation is often difficult to establish; mutations in different genes can present with similar phenotypes and different mutations in a single gene may result in different phenotypes.

Clinical implementation of massive parallel sequencing technology has emerged as a new approach to overcome the diagnostic complexity of myopathies and to reduce the necessity for invasive testing such as muscle biopsy. Correct and timely genetic diagnosis is critical for appropriate management of the patient, for prognostic information, and genetic counselling of the patient and his family as well as for carrier status determination and genetic diagnostics in prenatal settings. There is no widespread consensus for clinical indications for next generation sequencing (NGS), neither standardized NGS methodological approaches. Two approaches are mainly being implemented in clinical practice: exome or mendeliome sequencing and more frequently used targeted gene panel sequencing [3]. Few studies investigated the genetic diagnosis using NGS testing in unselected cohort of patients with myopathies. In recent years, there has been an increasing amount of studies conducted on subgroups of patients with a specific myopathy pattern, including patients with congenital muscular disorders, limb girdle muscular dystrophies or distal myopathies [4–14]. However, far less attention has been paid to the accuracy and efficiency of sequencing technology in unselected group of patients with various myopathy diagnoses [15–19].

There is a high variability in selection of NGS based diagnostic approaches for characterisation of patients with myopathies and the diagnostic yield obtained from the implementation of such methodologies varies significantly between studies [4–22]. Targeted gene panel sequencing approach was commonly used, but the selection of targeted genes differs greatly both in number and composition of genes [4–22].

The aim of the present study was to present the experience of systematic, routine use of novel NGS-based diagnostic approach addressing both clinical and genetic heterogeneity of myopathies. It is important to emphasize that we sought to assess the diagnostic yield and clinical utility of this approach in a group of patients who were consecutively referred to our centre with myopathy diagnosis, including patients with unspecified myopathy due to overlapping and non-specific clinical and laboratory findings.

## Patients and methods

### Patients

We conducted a retrospective study of patients with a clinical diagnosis of myopathy who were referred for a genetic evaluation and testing to the Clinical Institute of Medical Genetics, University Medical Centre, Ljubljana, Slovenia, in a period from 2014–2018. Patients with specific myopathic phenotype associated with specific mutation mechanisms like facioscapulohumeral muscle dystrophy, myotonic dystrophies and Duchenne/Becker muscle dystrophy were diagnosed directly by gene-targeted assays and were therefore excluded from further evaluation in this study.

We included 86 patients, who were referred with either congenital myopathy (CMyop), muscular dystrophy, congenital myotonia (CMyot), mitochondrial myopathy (MitM) or an unspecified myopathy (UM). Patients with muscular dystrophy were further categorised into limb girdle muscular dystrophy (LGMD), Duchenne/Becker MD (DMD) (without deletion/duplication diagnosed by multiplex ligation-dependent probe amplification) and distal myopathy (DM).

UM was defined when a clinical presentation was non-specific or incomplete, or when the clinical phenotype was suggestive for a myopathy, but EMG and muscle biopsy were not performed, were normal, or unspecific. Patients with UM were eligible for exome sequencing in a case of positive family history or paediatric age of symptom onset or when no other acquired cause of myopathy could be identified by a clinical diagnostic workup.

Patients were referred for genetic evaluation by a paediatric/adult neurologist. All patients received genetic counselling and were referred for exome sequencing by a clinical geneticist. Patients’ data were obtained through medical records review, including age of symptom onset, family history, clinical evaluation of muscle symptoms, and associated phenotypic features (e.g. facial dysmorphism, cardiomyopathy, cognitive delay) as well as standardised clinical diagnostic workup findings (examination by neurologist specialised in neuromuscular diseases, serum creatine kinase level, electromyography (EMG), and muscle biopsy). Phenotypic features were described according to the Human Phenotype Ontology (HPO) nomenclature [23].

### Ethics statement

In this study we analysed retrospectively the results of genetic testing previously performed as a part of a routine clinical diagnostics at our institution. No genetic testing was performed solely for the purpose of this study. All patients provided written informed consent. All patients were de-identified (regarding detected genetic variants data included in the manuscript). No other individual patient data was included.

### Exome sequencing, data analysis and interpretation

Sequencing was performed using a standardized series of procedures, starting with an in-solution capture of exome sequences (TruSight One, TruSight Exome, and Nextera Coding Exome capture kits, Agilent SureSelect Human All Exon v2, Agilent SureSelect Human All Exon v5 capture kits). This was followed by sequencing on Illumina MiSeq or Illumina HiSeq 2500 platform. The exome data analysis was completed using internally developed pipeline based on the combined disease and phenotype gene target definition approach, as we previously described [24,25]. Basic analysis, including SNV and indel discovery and annotation, was performed according to Genome Analysis Toolkit Best Practices workflow [26–29]. The Genome Aggregation Database (gnomAD) was employed as source of variant frequencies in worldwide populations. We also used Slovenian genomic database at our Institute which is planned to be an open database in the future. The details of diagnostic exome sequencing, analysis and interpretation were previously described [24,25]. When possible, family exome sequencing or variants segregation in families through Sanger sequencing was performed. All variants were classified according to the guidelines of the American College of Medical Genetics (ACMG) modified according to Association for Clinical Genomic Science (ACGS) recommendations as pathogenic, likely pathogenic, variant of uncertain significance (VUS), likely benign, or benign [27,28]. Evidence support level was weighted using modifiers VSTR (very strong), STR (strong), MOD (moderate) or SUP (supporting), where applicable according to ACGS recommendations [28]. Variant interpretations were submitted to the ClinVar Database (https://www.ncbi.nlm.nih.gov/clinvar/). Pathogenic and likely pathogenic variants were classified as disease causing variants.

### Gene selection

The search through OMIM (http://www.ncbi.nlm.nih.gov/omim) and Pubmed (http://www.ncbi.nlm.nih.gov/pubmed) databases enabled the creation of a 250 myopathy associated gene panel (S1 Table). The panel was supplemented with phenotype-based gene panel, which was generated using a Web tool (http://kimg.eu/generator). This tool enabled tracking for genes associated with clinical signs and symptoms using HPO nomenclature [24,25,30]. This database-based gene panel was applied for the filtration of sequence variants. In case of negative results, untargeted interpretation of all exome variants was performed by using internally developed variant analysis and interpretation pipeline.

## Results

### Patients

The study included 86 probands (38 males and 48 females). There were 22 paediatric and 64 adult patients. The mean age of the paediatric patients was 8.36 years (range from 36 months to 16 years) and the mean age of symptom onset was 16 months (range from birth to 7 years). In adult patients, the mean age was 41.22 (range from 18 to 72 years). The mean age of symptom onset in adult patients was 21.40 years (range from birth to 65 years). Nineteen cases (22%) had records of family history of the disease.

Groups of patients referred with diagnosis of CMyop, LGMD, DMD, CMyot, MitM and DM comprised 19 (22%), 10 (12%), 7 (8%), 5 (6%), 3 (3%), and 2 (2%) patients, respectively, altogether 46 patients. The remaining 40 patients (47%) were referred with the diagnosis of UM; 10 were paediatric cases (1 case with family history), and 30 were adults (9 cases with family history).

Overall, EMG was performed in 77% (66/86) and muscle biopsy in 66% (57/86) of referred patients.

EMG and muscle biopsy findings in patients with UM are presented in Table 1.

**Table 1:** EMG and muscle biopsy in paediatric and adult patients with an unspecified myopathy.

Patients with an unspecified myopathy	Paediatric patients Number (%)	Adult patients Number (%)
**Electromyography and muscle biopsy findings**		
Myopathic EMG and/or MB	6 (60)	27 (90)
Normal EMG / MB not performed	1 (10)	0 (0)
Normal on both examinations	0 (0)	1 (3)
Not performed	3 (30)	2 (7)
Total	10 (100)	30 (100)

MB–muscle biopsy; EMG–electromyography.

### Diagnosis by exome sequencing and the compatibility of genetic variant interpretation with the referral diagnosis

The diagnostic yield in our cohort of patients is presented in Table 2. Overall, genetic diagnosis was achieved in 52% (45/86) of cases.

**Table 2:** Diagnostic yield in patients grouped according to referral diagnosis.

Referral diagnosis	LGMD N (%)	CMyop N (%)	DMD N (%)	CMyot N (%)	MitM N (%)	DM N (%)	UM N (%)	Total N
Interpretation of genetic variant								
Pathogenic/likely pathogenic	7 (70)	9 (47)	7 (100)	5 (100)	1 (33)	2 (100)	14 (35)	45
Variant of uncertain significance	2 (20)	4 (21)	0 (0)	0 (0)	0 (0)	0 (0)	15 (38)	21
Likely benign /benign/ no variants detected	1 (10)	6 (32)	0 (0)	0 (0)	2 (67)	0 (0)	11 (28)	20
Total	10 (100)	19 (100)	7 (100)	5 (100)	3 (100)	2 (100)	40 (100)	86

LGMD—limb girdle muscular dystrophy; CMyop—congenital myopathy; DMD—Duchenne/Becker muscular dystrophy; CMyot—congenital myotonia; MitM–mitochondrial myopathy; DM—distal myopathy; UM—unspecified myopathy.

Among patients referred with a specific clinical myopathy pattern, pathogenic variants were identified in 67% (31/46) of cases. Clinical diagnosis was confirmed in 90% (28/31) of patients and in 10% of cases (3/31) genetic diagnosis revealed that myopathic symptoms were a part of syndrome or other rare neuromuscular disease (S2 Table). The diagnostic yield among patients referred with UM was 35% (14/40) (Table 2).

Overall, among 38 cases with a genetic diagnosis of myopathy, 32 (84%) had myopathic EMG and/or muscle biopsy, 1 had normal EMG (muscle biopsy was not performed), and in 5 patients neither EMG nor muscle biopsy were performed. In 7 patients with other rare pathogenic variants, 4 (57%) had myopathic EMG and/or muscle biopsy, 1 patient had normal EMG (muscle biopsy not performed), while in 2 patients neither EMG nor muscle biopsy were performed. Out of 20 cases with likely benign, benign or no variants detected, 17 (85%) had myopathic EMG and/or muscle biopsy, 2 had normal EMG (muscle biopsy not performed) and in 1 case neither EMG nor muscle biopsy were performed. There were also 21 cases with VUS variants, whereas 17 had myopathic EMG and/or muscle biopsy and 4 had normal findings or neither of the examinations were performed.

### Diagnostic rate in paediatric and adult cases

**Fig 1:**
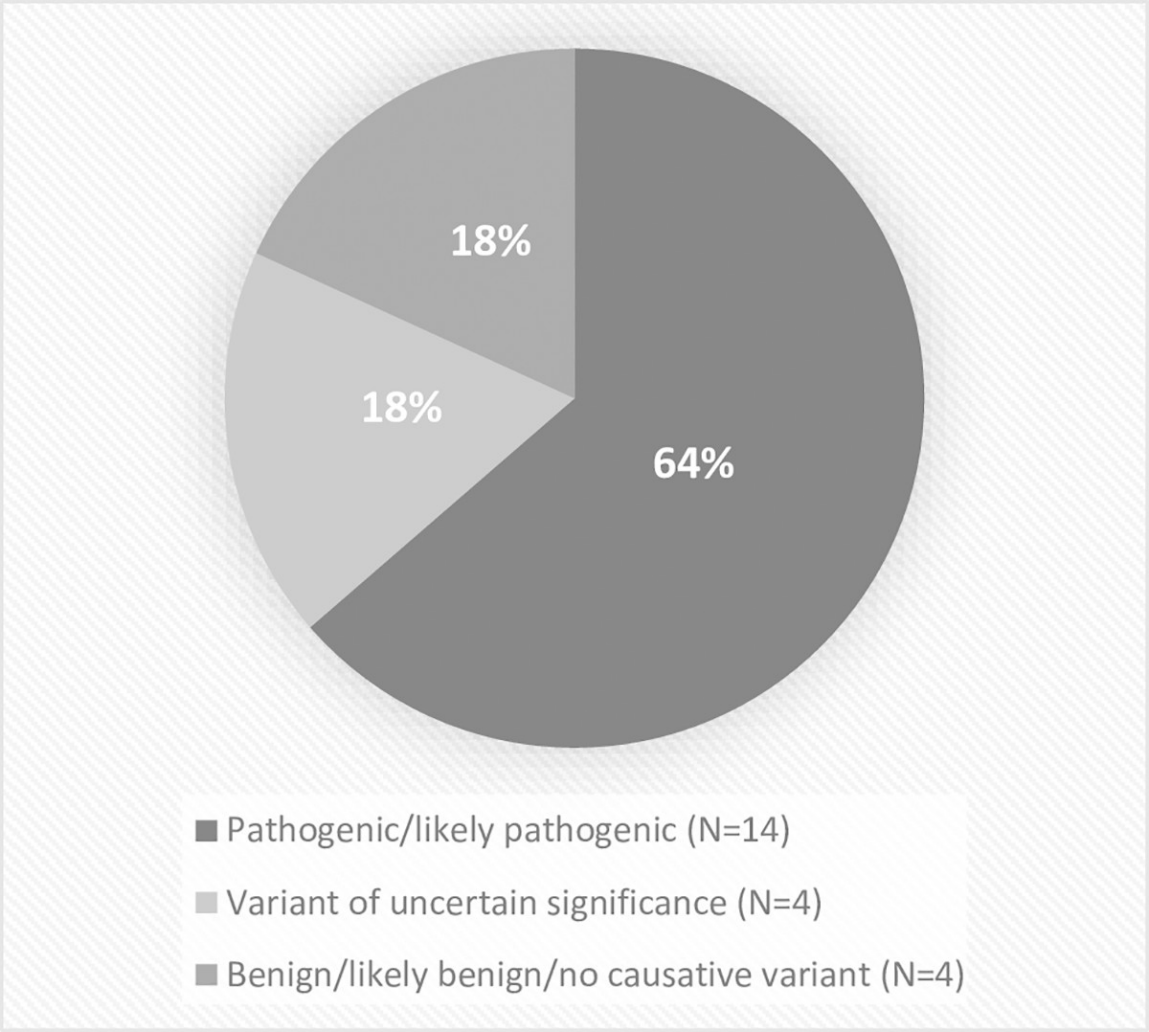
Interpretation of detected genetic variants in paediatric patients.

Among paediatric patients the two groups, DMD and CMyop, comprised 23% (5/22) of cases. Two patients were referred with LGMD. Almost a half (10/22, 45%) of cases were referred with UM. Overall, NGS enabled diagnosis in 64% (14/22) of paediatric cases (Fig 1); myopathy was confirmed in 79% (11/14) of probands and in 21% (3/14) of patients the final diagnosis revealed that myopathic symptoms were associated with other rare diseases (S2 Table). The average time from disease symptom onset to the genetic diagnosis was 7.05 years.

**Fig 2:**
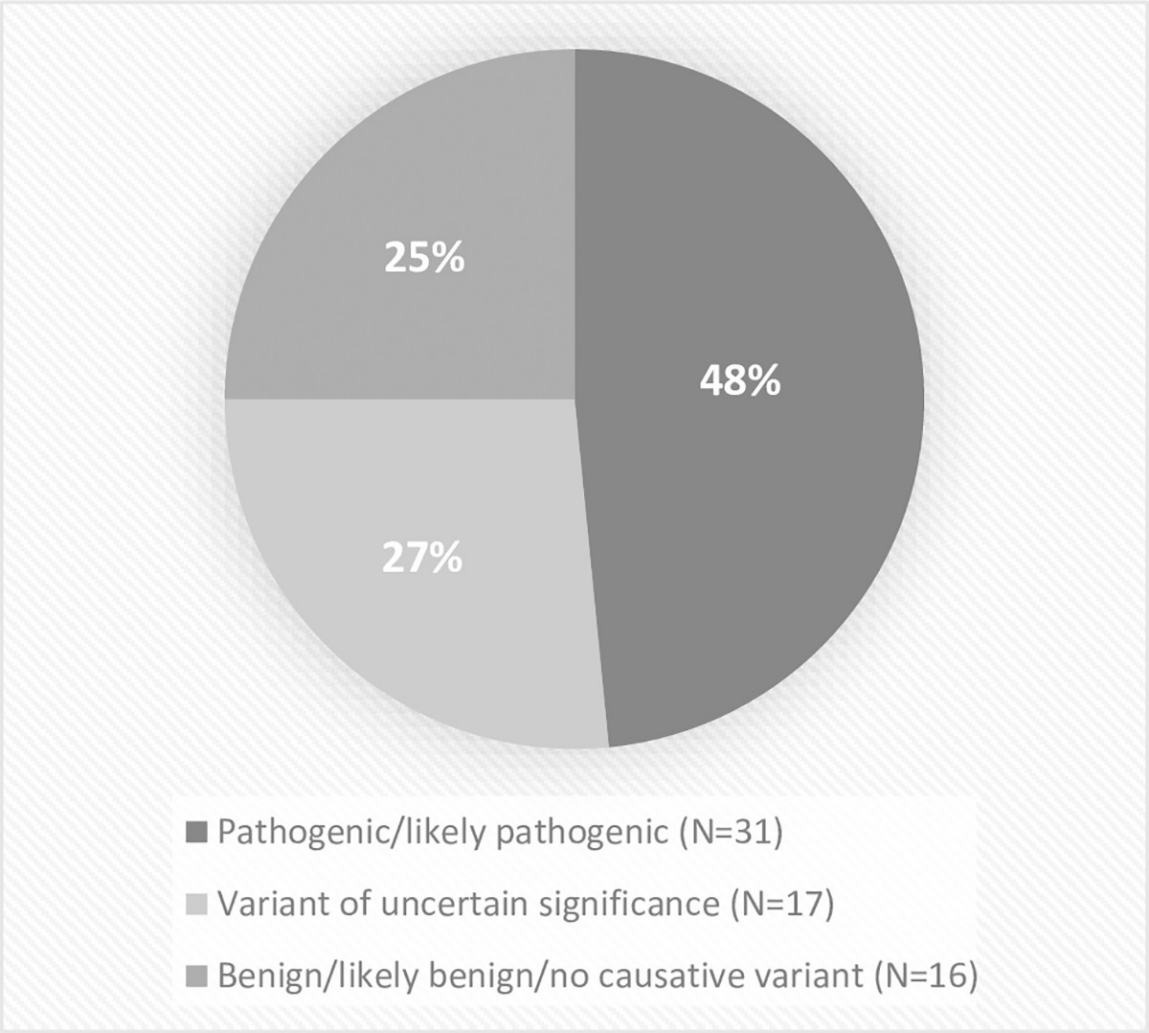
Interpretation of detected genetic variants in adult patients.

Adult patients were referred with UM, LGMD, CMyop and CMyot in 47% (30/64), 13% (8/64), 22% (14/64) and 8% (5/64) of cases, respectively. The remaining cases were referred with DMD (2/64), MitM (3/64) and DM (2/64). The diagnostic yield among adults was 48% (31/64) (Fig 2), where genetic diagnosis confirmed myopathy in 87% (27/31) of patients and in 13% of cases (4/31) genetic testing established other rare diseases with associated myopathic symptoms (S2 Table). The average time to genetic diagnose was 19.63 years.

### Classification and interpretation of identified genetic variants

Identified pathogenic /likely pathogenic variants for a myopathy are presented in Table 3. In total, 50 pathogenic/likely pathogenic variants were found in 22 nuclear genes and one variant was detected in the mitochondrial genome. Eight (15%) were novel. Two pathogenic copy number variations (CNV) were detected (duplication 22q11.2 and 17q12).

**Table 3:** Identified pathogenic/likely pathogenic variants.

Patient	Nucleotide Change	Zygosity/Inheritance	ACMG classification	Variant type	Protein Change	Theoretical predictions for missense or splice variants	gnomAD /MitoMap	SGDB	Citation	Novel variant
Reported disease gene: ACADVL										
P1	NM_000018.3: c.1358G>A	CHt AR	4 (PM3_STR, PM2, PP3)	Missense	p.Arg453Gln	Polyphen2: Probably damaging, Mutationtaster: Disease causing Automatic, Sift: Deleterious, Metasvm: D, Cadd 32.0, Revel: 0.9974, Gerp: 5.74	0.00001	0	ClinVar374123 PMID: 9973285, 32558070, 30194637, 20060901, 17999356, 7668252, 20301763, 10077518, 17374501, 8554073, 8845838, 9461620, 9546340, 9599005, 9839948	No
	NM_000018.3: c.1376G>A		5 (PM2, PM3_STR, PM5, PP3)	Missense	p.Arg459Gln	Polyphen2: Probably damaging, Mutationtaster: Disease causing Automatic, Sift: Tolerated, Metasvm: D, Cadd: 34.0, Revel: 0.9593, Gerp: 5.74	0.00002	0	ClinVar203585 LOVD:ACADVL_000048 PMID: 14517516, 19327992, 21429517, 23798014, 25214167 32906206	No
Reported disease gene: ACTA1										
P2	NM_001100.3: c.16G>A	Ht AD	4 (PS4, PM2, PP2, PP3, PP4)	Missense	p.Glu6Lys	Polyphen2: Benign, Mutationtaster: Disease causing, Sift: Deleterious, Metasvm: D, Cadd: 23.6, Revel: 0.92069, Gerp: 4.77	0	0	ClinVar42107 LOVD: ACTA1_000002 PMID: 19562689, 24642510, 26172852, 15468086	No
P3	NM_001100.3: c.593G>A	Ht AD	4 (PS4_SUP, PM2, PM5, PP2, PP3)	Missense	Arg198His	Polyphen2: Probably damaging, Mutationtaster: Disease causing, Sift: Deleterious, Metasvm: D, Cadd: 34.0, Revel: 0.97215, Gerp: 4.4	0	0	LOVD:ACTA1_000075 PMID: 19562689	No
Reported disease gene: ANO5										
P4	NM_213599.2: c.191dup	CHt AR	5 (PVS1, PM3, PP1)	Frameshift	Asn64LysfsTer15		0.0011	0.001	ClinVar2164 LOVD:ANO5_00004 PMID: 21186264, 23606453, 20096397, 21739273, 23607914, 32528171, 9673985, 21820307	No
	NM_213599.2: c.2029+2dup		4 (PM2, PM3, PP3, PP4)	Splice-site variant	/	VarSEAK Online Splice Prediction: Class 5—splicing effect	0	0	ClinVar374043	Yes
P5	NM_213599.2: c.1664G>T	CHt AR	4 (PM2, PM3_STR, PP4)	Missense	p.Ser555Ile	Polyphen2: Probably damaging, Mutationtaster: Disease causing, Sift: Deleterious, Metasvm: D, Cadd: 26.9, Revel: 0.72701, Gerp: 6.17	0.00006	0.002	ClinVar286450 LOVD:ANO5_000071 PMID: 25891276, 26886200, 27911336, 30564623, 31395899, 31341644, 31791368, 32528171	No
	NM_213599.2: c.1965G>C		4 (PM2, PM3, PP4, PP5)	Missense	p.Trp655Cys	Polyphen2: Probably damaging, Mutationtaster: Disease causing, Sift: Deleterious, Metasvm: D, Cadd: 33.0, Revel: 0.92601, Gerp: 5.71	0.00001	0	ClinVar523571 LOVD:ANO5_000064 PMID: 27447704, 22499103, 23047743	No
P6	NM_213599.2 c.191dup	CHt AR	5 (PVS1, PM3, PP1)	Frameshift	Asn64LysfsTer15		0.0011	0.001	ClinVar2164 LOVD:ANO5_00004 PMID: 21186264, 23606453, 20096397, 21739273, 23607914, 32528171, 9673985, 21820307	No
	NM_213599.2: c.2395C>T		4 (PVS1, PM2)	Premature stop codon insertion	p.Arg799Ter		0.00001	0	ClinVar930392 PMID:23606453	No
P7	NM_213599.2: c.191dup	Hom AR	5 (PVS1, PM3, PP1)	Frameshift	Asn64LysfsTer15		0.0011	0.001	ClinVar2164 LOVD:ANO5_00004 PMID: 21186264, 23606453, 20096397, 21739273, 23607914, 32528171, 9673985, 21820307	No
Reported disease gene: CAPN3										
P8 P9 P10	NM_000070.2: c.550del	Hom AR	5 (PVS1, PM2, PM3_VSTR, PP4)	Frameshift	p.Thr184fs		0.00022	0.007	ClinVar:17621 LOVD:CAPN3_000010 PMID: 7720071, 9266733,10330340, 10679950, 14578192, 15351423, 32528171, 33304817	No
Reported disease gene: CHAT										
P11	NM_020549.4: c.2081C>G	CHt AR	4 (PS3_SUP, PM2, PM3, PP1, PP3)	Missense	Ser694Cys	Polyphen2: Probably damaging, Mutationtaster: Disease causing, Sift: Deleterious, Metasvm: D, Cadd: 29.8, Revel: 0.99525, Gerp: 5.76	0	0	ClinVar523529 LOVD: CHAT_000012 PMID: 15701560, 12548525, 26080897, 29189923, 19688192, 15907919	No
	NM_020549.4: c.1061C>T		4 (PM2, PM3, PP1, PP3)	Missense	Thr354Met	Polyphen2: Probably damaging, Mutationtaster: Disease causing, Sift: Deleterious, Metasvm: D, Cadd: 25.7, Revel: 0.98621, Gerp: 5.35	0	0	LOVD: CHAT_000015 PMID: 15701560, 19520274, 29189923	No
Reported disease gene: COL6A1										
P12	NM_001848.2: c.814G>A	Ht AD	4 (PM1, PM2, PM5, PP3)	Missense	p.Gly272Ser	Polyphen2: Probably damaging, Mutationtaster: Disease causing Automatic, Sift: Deleterious, Metasvm: D, Cadd: 26.4 Revel: 0.99138, Gerp: 3.6	0	0	ClinVar93889 LOVD:COL6A1_00025 PMID: 29406609	No
Reported disease gene: COL6A2										
P13	NM_001849.3: c.1970-9G>A	Hom AR	5 (PS3, PM2, PM3_STR)	Splice-site variant	/	Ada Score 0.994089 Rf Score 0.938	0.00011	0	ClinVar265506 LOVD:COL6A2_00056 PMID: 19309692, 21280092, 25535305, 20576434, 29774307, 28578317, 15689448, 27447704, 24314752	No
Reported disease gene: CLCN1										
P14	NM_000083.2: c.2680C>T	Hom AR	5 (PVS1, PS3, PM3_STR)	Premature stop codon insertion	p.Arg894Ter		0.00288	0	ClinVar17545 LOVD: CLCN1_000163 PMID: 8533761, 11840191, 18337730, 8845168, 15162127, 22197187, 27614575, 26096614, 22094069, 7874130, 27296017, 27142102, 24349310, 15162127, 8533761, 17990293	No
P15	NM_000083.2: c.870C>G	Ht AD	5 (PS3, PS4_MOD, PM2, PP1_SUP, PP4)	Missense	p.Ile290Met	Polyphen2: Probably damaging, Mutationtaster: Disease causing Automatic, Sift: Deleterious, Metasvm: D, Cadd: 24.5, Revel: 0.96956, Gerp: 1.21	0	0	ClinVar17539 LOVD:CLCN1_00057 PMID: 7581380, 25036107, 25036107, 23739125, 12390967, 10051520, 10962018	No
P16	NM_000083.2: c.2635C>T	CHt AR	5 (PVS1, PM2, PM3_SUP, PP4)	Premature stop codon insertion	p.Gln879Ter		0.00001	0	ClinVar374131 LOVD: CLCN1_000224 PMID:23113340, 23739125	No
	NM_000083.2: c.501C>G		4 (PM2, PM3_STR, PP4)	Missense	p.Phe167Leu	Polyphen2: Benign, Mutationtaster: Disease causing, Sift: Tolerated, Metasvm: T, Cadd: 18.3, Revel: 0.8306, Gerp: 4.11	0.00117	0	ClinVar17016 LOVD: CLCN1_000022 PMID: 28993909, 8533761, 10690989, 22407275, 23933576, 24088041, 22641783, 26510092, 7874130, 23739125, 24304580, 18337730, 24452722, 22521272, 23113340, 21221019, 27199537, 22094069, 17654559, 22641783	No
Reported disease gene: DMD										
P17	NM_004006.2: c.5699T>G	Hem XR	5 (PVS1, PM2, PM3_SUP)	Premature stop codon insertion	p.Leu1900Ter		0	0	ClinVar374059 LOVD: DMD_001113	No
P18	NM_004006.2: c.1637G>A	Hem XR	5 (PVS1, PM2, PM3_SUP)	Premature stop codon insertion	p.Trp546Ter		0	0	ClinVar374191 LOVD:DMD_001151 PMID: 17253928, 21515508	No
P19	NM_004006.2: c.357+1G>A	Hem XR	4 (PVS1, PM2)	Splice-site variant	/	Gerp 5.44 Ada Score 0.99 Rf Score 0.93	0	0	ClinVar523467 LOVD:DMD_002071	No
P20	NM_004006.2: c.93+1G>C	Hem XR	5 (PVS1, PM2, PM3_SUP)	Splice-site variant	/	Gerp 5.23 Ada Score 0.999987 Rf Score 0.926	0	0	ClinVar 523470 LOVD:DMD_002270 PMID: 19937601	No
P21	NM_004006.2: c.10126del	Hem XR	5 (PVS1, PM2, PM3_STR)	Frameshift	p.Leu3376Ter		0	0	ClinVar287624 LOVD DMD_00063 PMID: 7581396, 17041906, 21969337, 27593222, 33101180	No
P22	NM_004006.2: c.4838G>A	Hem XR	5 (PVS1, PM2, PM3_MOD)	Premature stop codon insertion	p.Trp1613Ter		0	0	Clinvar523468 LOVD:DMD_002119 PMID: 31081998, 26968818	No
P23	NM_004006.2: c.177del	Ht XR	4 (PVS1, PM2)	Frameshift	p.Gly59fs		0	0	ClinVar374010	No
Reported disease gene: EMD										
P24	NM_000117.2: c.184dup	Hem XR	5 (PVS1, PM2, PP1)	Frameshift	p.Ser62fs		0	0	ClinVar: 523333 LOVD:EMD_000018 PMID: 11063761	No
Reported disease gene: GCH1										
P25	NM_000161.3: c.655C>T	Ht AD	4 (PVS1, PM2)	Premature stop codon insertion	p.Gln219Ter		0	0	ClinVar523338	Yes
Reported disease gene: GMPPB										
P26	NM_013334.3: c.684dup	CHt AR	4 (PVS1, PM2)	Frameshift	p.Met229fs		0	0	ClinVar 931364	Yes
	NM_013334.3: c.79G>C		4 (PM2, PM3, PP1, PP3)	Missense	p.Asp27His	Polyphen2: Probably damaging, Mutationtaster: Disease causing, Sift: Deleterious, Metasvm: D, Cadd: 25.6, Revel: 0.85586, Gerp: 4.84	0.00071	0	ClinVar60546 LOVD: GMPPB_000004 PMID: 27159402, 23768512, 25681410, 30060766, 32528171 26310427, 26133662, 25770200	No
Reported disease gene: LMNA										
P27	NM_170707.3: c.130G>T	Ht AD (dn)	4 (PS4_SUP, PM2, PM6, PP3)	Missense	p.Val44Phe	Polyphen2: Posibly damaging, Mutationtaster: Disease causing, Sift: Tolerated, Metasvm: T, Cadd: 24.8, Revel: 0.88401, Gerp: 2.83	0	0	ClinVar374201 LOVD:LMNA_00060	No
Reported disease gene: MT-TS2										
P28	NC_012920.1:m.12230A>G	Htp Mt	4*	Missence	/		0	0	/	Yes
Reported disease gene: MYOT										
P29 P30	NM_006790.2: c.164C>T	Ht AD	5 (PS3, PS4, PM2, PM5, PP3)	Missense	p.Ser55Phe	Mutationtaster: Disease causing Automatic, Sift: Deleterious, Metasvm: T, Cadd: 23.7, Revel: 0.63418, Gerp: 6.02	0	0	ClinVar5835 LOVD: MYOT_000003 PMID:12428213, 16684602, 9027924, 26342832, 21676617, 25208129, 15111675, 15947064, 21361873	No
Reported disease gene: PLOD1										
P31	NM_000302.3: c.1562G>A	CHt AR	4 (PVS1, PM2)	Premature stop codon insertion	p.Trp521Ter		0	0	ClinVar374077	Yes
	NC_000001.11:g.(11959822_11959973)_(11968469_11968718)dup		5 (PM2, PM3_VSTR, PP1)	Exon duplication	/		0	0	ClinVar14365 PMID: 8981946, 7977351, 8449506, 4373475, 5016372	No
Reported disease gene: RAPSN										
P32	NM_005055.4: c.264C>A	CHt AR	5 (PS3, PM2, PM1, PM3, PP3)	Missense	Asn88Lys	Polyphen2: Probably damaging, Mutationtaster: Disease causing Automatic, Sift: Deleterious, Metasvm: D, Cadd: 28.3, Revel: 0.69425, Gerp: 5.14	0.00156	0.002	ClinVar8046 LOVD: RAPSN_000002 PMID: 12796535, 16945936, 14504330, 11791205, 12807980, 12929188, 14729848	No
	NM_005055.4: c.490C>T		5 (PS3, PM2, PM3, PP4, PP3)	Missense	Arg164Cys	Polyphen2: Probably damaging, Mutationtaster: Disease causing Automatic, Sift: Deleterious, Metasvm: T, Cadd: 29.7, Revel: 0.70014, Gerp: 5.14	0.00002	0	ClinVar8054 LOVD: RAPSN_000035 PMID: 16931511, 32528171, 32070632, 27966543	No
Reported disease gene: RYR1										
P33	NM_000540.2: c.7111G>A	Ht AD (dn)	5 (PS3, PS4, PM2, PM6, PP3)	Missense	p.Glu2371Lys	Polyphen2: Probably damaging, Mutationtaster: Disease causing, Sift: Deleterious, Metasvm: D, Cadd: 26.9, Revel: 0.96919, Gerp: 3.99	0	0	ClinVar374164 LOVD:RYR1_000997 PMID: 29293505, 19191333	No
P34	NM_000540.2: c.3535C>T	CHt AR	4 (PM3, PM2, PP3, PP4)	Missense	p.Arg1179Trp	Polyphen2: Probably damaging, Mutationtaster: Benign, Sift: Deleterious, Metasvm: T, Cadd: 28.9, Revel: 0.82724, Gerp: 2.86	0.00001	0	ClinVar374168 PMID: 22473935, 25370123	No
	NM_000540.2: c.11315G>A		4 (PM2, PM3, PM5, PP1, PP3)	Missense	p.Arg3772Gln	Polyphen2: Probably damaging, Mutationtaster: Disease causing, Sift: Deleterious, Metasvm: D, Cadd: 27.7, Revel: 0.9681, Gerp: 4.82	0.00002	0	ClinVar133012 LOVD:RYR1_000295 PMID: 17483490, 19645060, 18253926, 33767344	No
P35	NM_000540.2: c.9579C>G	CHt AR	4 (PM2, PM3, PP3, PS3_Sup)	Missense	Cys3193Trp	Polyphen2: Probably damaging, Mutationtaster: Disease causing, Sift: Deleterious, Metasvm: D, Cadd: 27.8, Revel: 0.95499, Gerp: 3.75	0	0	ClinVar159864 LOVD:RYR1_00417 PMID: 22473935, 31680123	No
	NM_000540.2: c.8463G>A		4 (PVS1, PM2)	Premature stop codon insertion	p.Trp2821Ter		0	0	ClinVar373926	Yes
Reported disease gene: SCN4A										
P36	NM_000334.4 c.1333G>T	Ht AD	4 (PS4, PM2, PM5, PP3)	Missense	p.Val445Leu	Polyphen2: Probably damaging, Mutationtaster: Disease causing, Sift: Deleterious, Metasvm: D Cadd: 23.5, Revel: 0.96117, Gerp: 4.9	0	0	ClinVar373945 LOVD:SCN4A_000225 PMID: 32849172, 31567646, 33263785	No
Reported disease gene: SGCA										
P37	NM_000023.2: c.229C>T	Hom AR	5 (PM2, PP3, PM3_STR, PS3)	Missense	p.Arg77Cys	Polyphen2: Probably damaging, Mutationtaster: Disease causing Automatic, Sift: Deleterious, Metasvm: D, Cadd 34.0, Revel: 0.99072, Gerp: 4.53	0.0005	0	ClinVar9437 LOVD:SGCA_000003 PMID: 8528203, 7663524, 7657792, 8866424, 9032047, 12746421, 15736300, 21856579, 27120200	No
Reported disease gene: SYT2										
P38	NM_001136504.1 c.1084_1089delTATGAC	Ht AD (dn)	4 (PM2, PM4, PM6, PP4)	In-frame deletion	Tyr362_Asp363del		0	0	ClinVar 373966	Yes
Reported disease gene: TRIM32										
P39	NM_001099679.1 c.1459G>A	Hom AR	5 (PS3,PM2, PM3, PP1, PP3)	Missense	p.Asp487Asn	Polyphen2: Probably damaging, Mutationtaster: Disease causing Automatic, Sift: Deleterious, Metasvm: D, Cadd: 29.4, Revel: 0.81818, Gerp: 5.47	0.00002	0.001	ClinVar7350 LOVD: TRIM32_000001 PMID: 23142638, 15786463,11822024, 21775502	No
Reported disease gene: TTN										
P40^R^ P41^R^	NM_001267550.2:c.107635C>T	CHt AR	5 (PVS1, PM2, PM3)	Premature stop codon insertion	p.Gln35879Ter		0.00001	0.002	ClinVar202529 LOVD:TTN_001009 PMID: 28295036, 27796757, 29435569, 32528171	No
	NM_001267550.2:c.103360del		5 (PVS1, PM2, PM3)	Premature stop codon insertion and frameshift	p.Glu34454Asnfs		0.00002	0	ClinVar374145 LOVD: TTN_001008 PMID: 28295036, 18948003, 23975875, 24395473, 25589632	No
P42	NM_001267550.2 c.47961del	CHt AR	4 (PVS1, PM2)	Frameshift	/		0	0	ClinVar523430	Yes
	NM_001267550.2 c.52102+5G>A		4 (PM2,PM3, PP3,PP4)	Splice-site variant	p.Thr15987fs	Ada Score 0.93253 Rf Score 0.594	0	0	ClinVar931929 PMID: 26841830	No
P43	NM_001267550.2 c.107635C>T	Hom AR	5 (PVS1, PM2, PM3)	Premature stop codon insertion	p.Gln35879Ter		0.00001	0.004	ClinVar202529 LOVD: TTN_001009 PMID: 28295036, 27796757, 29435569, 32528171	No

* could not be categorised according to ACMG as there are no guidelines for the mitochondrial variants. The absence of the variant from MitoMap database of mitochondrial variability, heteroplasmy of the variant and reported nearby pathogenic variant, m.12207G> A, in the case of a patient with MELAS / MERRF-like syndrome (PMID:16950817) supported its pathogenicity. XR: X-linked recessive; AD: Autosomal dominant; AR: Autosomal recessive; CHt: Compound heterozygosity; Ht: Heterozygosity; Hom: Homozygosity; Hem: Hemizygosity; Mt: Mitochondrial; R-related patients; SGDB: Slovenian genomic database.

Out of 42 patients with pathogenic genetic variants in nuclear genes, 11 carried heterozygous variants resulting in dominant type of a myopathy. Among these, 3 variants were of de novo origin, whereas the remaining pedigrees were consistent with dominant inheritance. A homozygous or compound heterozygous pathogenic variant was identified in 23 probands resulting in a recessive type of a myopathy. A hemizygous pathogenic variant was detected in 8 probands resulting in X-linked type of a myopathy.

Pathogenic CNVs and pathogenic/likely pathogenic variants in genes, including PLOD1, GCH1, CHAT, RAPSN, and SYT2 enabled the diagnosis of other rare diseases with myopathic symptoms (S2 Table).

## Discussion

Using a phenotype driven exome analysis diagnostic approach we demonstrated a high diagnostic yield of 52% in a cohort of patients who were consecutively referred to our centre with clinical signs and symptoms consistent with a myopathy. Our cohort of patients was not selected according to specific clinical pattern of myopathy and cases with UM were also included. To the best of our knowledge, the evaluation of clinical utility of NGS-based approach in patients with UM has not been conducted so far.

A growing body of literature has been investigating the benefits and the challenges of NGS based technology implementation in the standard clinical practice of patients with inherited myopathies [5–22]. So far, however, fewer studies have been conducted on patients with unselected primary muscular disease, whereas reported diagnostic yield ranged between 16% and 36% [15–19]. Park et al. included only patients, who presented with a specific clinical myopathy pattern [14]. Using targeted gene panel NGS diagnostic approach they obtained the diagnostic yield of 36%. Bugiardini et al. applied focused exome sequencing to investigate complex adult myopathy patients, which were categorized based on the age of symptom onset and predominant pattern of weakness [16]. Pathogenic or likely pathogenic variants were identified in 32% of cases. Haskel et al. performed exome sequencing in 93 undiagnosed paediatric and adult patients with various neuromuscular diseases, including 31 patients with myopathy phenotype, 21 patients with neuropathy phenotype and 41 complex patients with neuropathy, myopathy, and additional phenotype [17]. They used targeted gene panel (myopathy or neuropathy) and a broad neuromuscular gene panel. Among patients with myopathy phenotype diagnostic yield was 16.1%, regardless of the used gene panel. In a group of patients with a complex phenotype, they illustrated higher diagnostic rate (9.8%) using a broader diagnostic gene panel compared with using neuropathy (4.9%) or myopathy (0%) diagnostic gene panel alone. Punetha et al. carried out targeted sequencing with a myopathy candidate gene panel in a cohort of 94 undiagnosed muscle disease patients and obtained the diagnostic yield of 35% [18]. In recent study, Thuriot et al. used gene panel approach in a large cohort of patients with suspected muscle disorders and reported an overall diagnostic yield of 15.1% (22.4% in children and 13.7% in adults) [19]. Most of other reported NGS based studies focused on one or more types of myopathy, including congenital muscular disorder [5,8,11,13], LGMD [5,6,8,10,12,14], or DM [7,12]. Using targeted NGS approach [5–8] or WES [10–14] the reported diagnostic rate ranged from 12% to 52%, except for the study of Chinese patients presenting with muscular dystrophy and congenital myopathy, which demonstrated the diagnostic yield of 65% [9]. Our results showed a high diagnostic rate among cases with specific clinical pattern of myopathy (67%), whereas CMyop and LGMD represented the largest groups of patients (Table 2).

However, it was particularly interesting to assess the diagnostic yield and clinical utility of our exome sequencing approach in patients with UM, since our cohort encompassed 47% of such cases. Considering that myopathies are characterized by highly variable, non-specific clinical presentation and frequently overlapping phenotypes between different diseases, it is expected that some of the patients could not be designated with certainty into any of the myopathy subgroup. We were able to detect a pathogenic variant in 35% of patients with UM, which is in the range of published diagnostic rates obtained among patients with specific clinical pattern of muscular disease.

In addition, 26% of cases in our study were paediatric patients. A separate analysis of paediatric cases with myopathies revealed a high diagnostic yield of 64%, whereas the average time to diagnosis was 7.05 years. Our diagnostic rate coincides well with the results published by Schofield et al, which was conducted on 56 paediatric patients, referred with the diagnosis of congenital muscular dystrophy or nemaline myopathy [21]. Using the traditional investigation based on muscle biopsy and protein-based studies of muscle biopsy specimens, followed by candidate gene sequencing, they established the diagnosis in 46% of referred patients. They increased their diagnostic efficacy using neuromuscular gene panel to 75% and to 79% using WES. In recent study, WES analysis was conducted on 50 patients with undiagnosed paediatric‐onset neuromuscular diseases resulting with the overall diagnostic yield of 26% [22]. Diagnostic rate among patients with hereditary congenital myopathy was 17% and among patients with hereditary muscular dystrophy subgroup was 45%.

The diagnostic yield among our adult patients was also high (48%) and the average time to diagnose was 19.63 years.

Our approach also included the assessment of mitochondrial and CNV variations, which enabled the diagnosis of microduplication syndrome 17q12 in paediatric patient, referred with UM. Clinical variability of 17q12 duplication syndrome is broad and myopathy was already described in those patients [31]. Similarly, we revealed a likely causative 22q11.2 microduplication in a patient with predominant presentation of hypotonia and motor delay. As known microduplication 22q11.2 may manifest predominantly with severe muscle hypotonia (OMIM#608363). In addition, in one patient who presented with unspecific muscle symptoms, genetic diagnosis revealed Ehlers-Danlos type 4. Interestingly, our results also demonstrated that diagnostic exome sequencing may affect medical management of the patients. Genetic testing revealed Dopa-Responsive Dystonia (DRD) in a case referred as UM, as well as congenital myasthenic syndromes in 3 cases referred with UM or CMyop. Those patients started with symptomatic therapy after genetic diagnosis. These findings further confirm the added value of our exome sequencing approach in diagnostics of cases with unspecific myopathy phenotypes and further decipherment whether myopathic symptoms are a part of a syndrome or separate clinical feature.

Despite the accumulating evidence that NGS technology has high clinical utility and huge potential to become the first approach in clinical laboratories, there are no universally or widely accepted guidelines for genetic testing, regarding the patient selection or diagnostic approach in heterogeneous disorders. This is also because the selection of appropriate target genes is challenging and carries a risk of missing causative variation due to their restrictiveness, whereas time and cost efficiency of such approach is arguable [24]. In view of limitations of targeted testing, we have applied clinical exome sequencing supplemented with panel of genes associated with clinical signs/symptoms using HPO nomenclature. This enabled the assessment of patients in regard to clinical diagnostic hypothesis as well as presenting signs/symptoms. This approach has been particularly useful in patients with complex, atypical clinical presentation that does not direct the clinical diagnosis toward a specific disease, whereas some of those patients would not even be considered for genetic testing due to unfulfilled clinical criteria for a specific myopathy. We suggest that patients with specific clinical myopathy pattern as well as patients with UM and suspected genetic aetiology should be referred for genetic testing. Genetic diagnosis is of great importance for both, patients and their families, as it can have a significant impact on patient management (particularly in cases of treatable disorders), prognosis, accurate genetic counselling, prenatal diagnostics and avoiding additional diagnostic testing. Thus, the need for widely accepted recommendations, which will improve the diagnostic implementation of exome sequencing in clinical practice of heterogeneous disorders, still remains.

The strength of our study is the patient cohort, which also included cases with UM and suspected genetic aetiology. Secondly, our approach is independent of specificity and accuracy of the clinical diagnostic hypothesis. The study’s limitation is the number of patients with a particular muscular disorder, which hinders the evaluation of our exome sequencing diagnostic utility of rare disorders.

In conclusion, our results provide evidence that phenotype driven exome analysis diagnostic approach improves the diagnostic rate of complex, heterogeneous disorders, such as myopathies, particularly in patients with an unspecified myopathy.

## Supporting information

S1 Table 250 inherited muscular disorders associated gene panel. (DOCX)

S2 Table Characteristics of patients with disease-causing variants. LGMD—limb girdle muscular dystrophy; CMyop—congenital myopathy; DMD–Duchenne /Becker muscular dystrophy; CMyot—congenital myotonia; MitM–mitochondrial myopathy; DM—distal myopathy; UM—unspecified myopathy; N–negative, P–positive, M–male, F–female); XR: X-linked recessive; AD: Autosomal dominant; AR: Autosomal recessive, Mt–mitochondrial.
(DOCX)
