# Supplementary material for: Diagnostic yield of exome sequencing in myopathies: Experience of a Slovenian tertiary centre
Source: PLoS One. 2021 Jun 9;16(6):e0252953. doi: 10.1371/journal.pone.0252953 (PMC8189452; doi:10.1371/journal.pone.0252953)
Supplement: S1 Table — (DOCX) [file pone.0252953.s001.docx]

Supplemental table 1: 250 genes in miopathy associated gene panel

ACTA1, ACVR1, BIN1, CCDC78, CFL2, CHGB, CNTN1, ISCU, KBTBD13, LAMP2, MAMLD1, MEGF10, MSTN, MTM1, MTMR14, MT-TS2, MYF6, MYH2, PABPN1, PANK2, PFN1, PLEC, PON1, PON2, PON3, PRPH2, RYR1, RYR2, SEPN1, TNNT1, TPM2, TPM3, TRIM32, TTN, ANO5, CAV3, CRYAB, DES, DYSF, FHL1, FLNC, GNE, KLHL9, LDB3, MATR3, MYH14, MYH7, MYOT, NEB, TCAP, TIA1, VCP, ATP2A1, CACNA1S, CLCN1, COL6A1, COL6A2, COL6A3, DMD, GLRA1, GLRB, HSPG2, KCNA1, KCNE3, KCNJ18, KCNJ2, KCNQ2, SCN4A, DNM2, BAG3, ACAD9, ACADL, ACADM, ACADVL, AGL, AMPD1, C10orf2, CPT1B, CPT2, ETFA, ETFB, GAA, GYS1, HADHA, HADHB, LPIN1, OPA1, OPA3, PFKM, PGAM2, PGM1, PHKA1, POLG, POLG2, PYGM, RRM2B, SUCLA2, TK2, TYMP, ACADS, PYGM1, CAPN3, CNBP, DMPK, EMD, FKRP, FKTN, FXN, HRAS, LAMA2, LMNA, SGCD, SLC25A4, TAZ, TMEM43, TMPO, TNNI3, KLHL40, ABHD5, AMACR, ETFDH, FDX1L, HADH, PGK1, PNPLA2, SLC22A5, AARS, AGK, AGRN, ALG14, ALG2, B3GALNT2, B4GAT1, BICD2, CHAT, CHKB, CHRNA1, CHRNB1, CHRND, CHRNE, COL12A1, COLQ, COX15, CPT1A, CUL4B, DNAJB6, DOK7, DPAGT1, DPM1, DPM2, DPM3, DYNC1H1, EGR2, FKBP14, GARS, GBE1, GDAP1, GFM1, GFPT1, GMPPB, GOSR2, HINT1, HNRNPU, IGHMBP2, ISPD, ITGA7, KIF21A, LARGE, LAS1L, LIMS2, LRP4, MFN2, MICU1, MPZ, MTO1, MUSK, MYBPC1, NEFL, ORAI1, PHOX2B, PIEZO2, POMGNT1, POMGNT2, POMK, POMT1, POMT2, PREPL, PTRF, PUS1, RAPSN, SCO2, SDHA, SGCA, SGCB, SGCG, SIL1, SLC25A20, SLC25A3, SLC52A3, SLC6A5, SMCHD1, SNAP25, STAC3, STIM1, SYNE1, SYNE2, TMEM5, TMEM70, TNPO3, TNXB, TOR1AIP1, TRAPPC11, TRPV4, TTR, TUBB3, UBA1, KLHL41, COL9A3, GSN, NEBN, ALMS1, ARL6, BBS1, BBS10, BBS12, BBS2, BBS4, BBS5, BBS7, BBS9, CEP290, GNAS1, MKKS, MKS1, PHF6, SDCCAG8, TMEM67, TTC8, WDPCP, SEPT9, B3GNT1, DAG1, GTDC2, SGK196, ST3GAL4, ATP7A, PSMB8, VRK1, FRKP, CACNA1A.
