# Supplementary material for: Diagnostic yield of exome sequencing in myopathies: Experience of a Slovenian tertiary centre
Source: PLoS One. 2021 Jun 9;16(6):e0252953. doi: 10.1371/journal.pone.0252953 (PMC8189452; doi:10.1371/journal.pone.0252953)
Supplement: S2 Table — LGMD—limb girdle muscular dystrophy; CMyop—congenital myopathy; DMD–Duchenne /Becker muscular dystrophy; CMyot—congenital myotonia; MitM–mitochondrial myopathy; DM—distal myopathy; UM—unspecified myopathy; N–negative, P–positive, M–male, F–female); XR: X-linked recessive; AD: Autosomal dominant; AR: Autosomal recessive, Mt–mitochondrial. (DOCX) [file pone.0252953.s002.docx]

Supplemental table 2. Characteristics of patients with other rare disease-causing variants

| Patient | Referral  diagnosis | Main clinical  symptoms  (HPO IDs) | Age at onset | Sex | Familial anamnesis | Reported  disease gene | Inheritance | Genetic  diagnosis |
| --- | --- | --- | --- | --- | --- | --- | --- | --- |
| P1 | UM | Myopathy (HP:0003198), Rhabdomyolysis (HP:0003201), Exercise-induced muscle cramps (HP:0003710), Exercise-induced myalgia (HP:0003738). | 17 | M | N | ACADVL | AR | Very long-chain acyl-CoA dehydrogenase deficiency (OMIM:201475) |
| P2 | CMyop | Generalized hypotonia (HP:0001290), Generalized muscle weakness (HP:0003324), Muscle weakness (HP:0001324). | 0-1 | F | N | ACTA1 | AD | Myopathy, congenital, with fiber-type disproportion 1 (OMIM:255310) |
| P3 | CMyop | High palate (HP:0000218), Muscle weakness (HP:0001324) , Abnormality of the Achilles tendon (HP:0005109), Generalized hypotonia (HP:0001290), Motor delay (HP:0001270). | 0-1 | F | N | ACTA1 | AD | Myopathy, congenital, with fiber-type disproportion 1 (OMIM:255310), Nemaline myopathy 3 (OMIM:161800) |
| P4 | UM | Skeletal muscle atrophy (HP:0003202), Muscle weakness (HP:0001324), Proximal muscle weakness in lower limbs (HP:0008994). | 58 | M | N | ANO5 | AR | Miyoshi muscular dystrophy 3 (OMIM:613319), Muscular dystrophy, limb-girdle, autosomal recessive 12 (OMIM:611307) |
| P5 | UM | Fatty replacement of skeletal muscle Miopatija (HP:0003198), Distal muscle weakness (HP:0002460), Elevated serum creatine kinase (HP:0003236), EMG abnormality (HP:0003457). | 23 | M | N | ANO5 | AR | Miyoshi muscular dystrophy 3 (OMIM:613319), Muscular dystrophy, limb-girdle, autosomal recessive 12 (OMIM:611307) |
| P6 | UM | Myalgia (HP:0003326), Headache (HP:0002315), Elevated serum creatine kinase (HP:0003236), Diplopia (HP:0000651), Elevated hepatic transaminase (HP:0002910), Difficulty climbing stairs (HP:0003551), Muscle weakness (HP:0001324), Hand tremor rok (HP:0002378), Tinnitus (HP:0000360). | 33 | M | N | ANO5 | AR | Miyoshi muscular dystrophy 3 (OMIM:613319), Muscular dystrophy, limb-girdle, autosomal recessive 12 (OMIM:611307) |
|  |  |  |  |  |  |  |  |  |
| P7 | UM | Achilles tendon contracture (HP:0001771), Elevated serum creatine kinase (HP:0003236), Lower limb amyotrophy (HP:0007210), Lower limb muscle weakness (HP:0007340). | 31 | M | P | ANO5 | AR | Miyoshi muscular dystrophy 3 (OMIM:613319), Muscular dystrophy, limb-girdle, autosomal recessive 12 (OMIM:611307) |
| P8 | DMD | Congenital muscular dystrophy (HP:0003741). | 9 | M | P | CAPN3 | AR | Muscular dystrophy, limb-girdle, autosomal recessive 1 (OMIM: 253600) |
| P9 | LGMD | Shoulder girdle muscle weakness (HP:0003547), Calf muscle hypertrophy (HP:0008981). | 5 | F | N | CAPN3 | AR | Muscular dystrophy, limb-girdle, autosomal recessive 1 (OMIM: 253600) |
| P10 | LGMD | Limb-girdle muscle weakness (HP:0003325), Shoulder girdle muscle weakness (HP:0003547), Muscular dystrophy (HP:0003560). | 6 | F | N | CAPN3 | AR | Muscular dystrophy, limb-girdle, autosomal recessive 1 (OMIM: 253600) |
| P11 | UM | Myopathy (HP:0003198), Progressive ptosis (HP:0000508), Ophthalmoplegia (HP:0000602), Gastroesophageal reflux (HP:0002020), Pes planus (HP:0001763), Respiratory insufficiency (HP:0002093), Progressive muscle weakness (HP:0003323), Decreased activity of the pyruvate dehydrogenase complex (HP:0002928), Lactic acidosis (HP:0003128). | 1 | M | N | CHAT | AR | Myasthenic syndrome, congenital, 6, presynaptic (OMIM: 254210) |
| P12 | UM | Motor delay (HP:0001270), Limb-girdle muscle weakness (HP:0003325), EMG abnormality (HP:0003457). | 0-1 | F | P | COL6A1 | AD | Bethlem myopathy 1 (OMIM:158810), Ullrich congenital muscular dystrophy 1 (OMIM:254090) |
| P13 | CMyop | Congenital hip dislocation (HP:0001374), Hip flexor weakness (HP:0012515), Difficulty walking (HP:0002355), Falls (HP:0002527), Muscle weakness (HP:0001324). | 1 | F | N | COL6A2 | AR | Bethlem myopathy 1 (OMIM:158810), Ullrich congenital muscular dystrophy 1 (OMIM:254090) |
| P14 | CMyot | Myopathy (HP:0003198), EMG: myopathic abnormalities (HP:0003458), Myotonia (HP:0002486). | 12 | M | N | CLCN1 | AR | Myotonia congenita, recessive  (OMIM: 255700) |
| P15 | CMyot | Myotonia (HP:0002486), EMG: myotonic discharges (HP:0100284). | 14 | M | N | CLCN1 | AD | Myotonia congenita, dominant OMIM:160800 |
| P16 | CMyot | Myotonia (HP:0002486), EMG: myotonic discharges (HP:0100284). | 36 | F | N | CLCN1 | AR | Myotonia congenita, recessive  (OMIM: 255700) |
| P17 | DMD | Difficulty climbing stairs (HP:0003551), Calf muscle hypertrophy (HP:0008981), Gowers sign (HP:0003391). | 5 | M | N | DMD | XR | Duchenne muscular dystrophy  (OMIM: 310200) |
| P18 | DMD | Motor delay (HP:0001270), Proximal muscle weakness (HP:0003701), Calf muscle hypertrophy (HP:0008981). | 1 | M | N | DMD | XR | Duchenne muscular dystrophy  (OMIM: 310200) |
| P19 | DMD | Muscular dystrophy (HP:0003560), Flexion contracture (HP:0001371), Respiratory insufficiency (HP:0002093), Progressive muscle weakness (HP:0003323), Motor delay (HP:0001270). | 2 | M | N | DMD | XR | Duchenne muscular dystrophy  (OMIM: 310200) |
| P20 | DMD | Muscular dystrophy (HP:0003560),  Elevated serum creatine kinase (HP:0003236), Proximal muscle weakness (HP:0003701), Calf muscle hypertrophy (HP:0008981). | 5 | M | N | DMD | XR | Duchenne muscular dystrophy  (OMIM: 310200) |
| P21 | DMD | Motor delay (HP:0001270), Proximal muscle weakness in lower limbs (HP:0008994), Calf muscle hypertrophy (HP:0008981). | 1 | M | N | DMD | XR | Duchenne muscular dystrophy  (OMIM: 310200) |
| P22 | DMD | Muscular dystrophy (HP:0003560), Progressive muscle weakness (HP:0003323), Calf muscle hypertrophy (HP:0008981), Dilated cardiomyopathy (HP:0001644). | 1 | M | N | DMD | XR | Duchenne muscular dystrophy  (OMIM: 310200) |
| P23 | LGMD | Pelvic girdle muscle atrophy (HP:0008988), Exercise-induced muscle fatigue (HP:0009020), Elevated creatine kinase after exercise (HP:0008331), EMG abnormality (HP:0003457), Muscle weakness (HP:0001324), Exercise-induced muscle stiffness (HP:0008967), Exercise-induced muscle cramps (HP:0003710), Exercise-induced myalgia (HP:0003738), Exercise-induced rhabdomyolysis (HP:0009045). | 6 | F | N | DMD | XR | Duchenne muscular dystrophy  (OMIM: 310200) |
| P24 | LGMD | Flexion contracture (HP:0001371), Elevated serum creatine kinase (HP:0003236), Diplopia (HP:0000651), Elevated hepatic transaminase (HP:0002910), Scapular winging (HP:0003691), Myopathy (HP:0003198). | 1 | M | P | EMD | XR | Emery-Dreifuss muscular dystrophy 1 (OMIM:310300) |
| P25 | UM | Muscular weakness (HP:0001324), Difficulty walking (HP:0001288), Sleep disturbance (HP:0002360), Fatigue (HP:0012378), Difficulty walking (HP:0001288), Talipes equinovarus (HP:0001762), Lower limb muscle weakness (HP:0007340), Rigidity (HP:0002063), Imbalance walking (HP:0002141). | 0-1 | F | N | GCH1 | AD | Dopa-responsive dystonia (DRD, OMIM:128230), |
| P26 | CMyop | Muscular dystrophy (HP:0003560),  Proximal muscle weakness (HP:0003701), Limb pain (HP:0009763). | 16 | M | N | GMPPB | AR | Muscular dystrophy-dystroglycanopathy (limb-girdle), type A, B, C, 14 (OMIM:615350, 615351, 615352). |
| P27 | CMyop | Congenital muscular dystrophy (HP:0003741), Difficulty walking (HP:0002355), Muscle weakness (HP:0001324), EMG abnormality (HP:0003457), Muscular hypotonia (HP:0001252). | 0-1 | F | N | LMNA | AD | Muscular dystrophy, congenital (OMIM:613205) |
| P28 | MitM | Abnormality of mitochondrial metabolism (HP:0003287), Exercise intolerance (HP:0003546), Exercise-induced myalgia (HP:0003738), Increased serum lactate (HP:0002151), Mitochondrial myopathy (HP:0003737), Ptosis (HP:0000508). | 10 | M | N | MT-TS2 | Mt | OMIM:590085, PMID:16950817 (MELAS) |
| P29 | DM | Distal amyotrophy (HP:0003693), Distal lower limb muscle weakness (HP:0009053), EMG: myopathic abnormalities (HP:0003458), Foot dorsiflexor weakness (HP:0009027), Lower limb pain (HP:0012514), Muscle fiber inclusion bodies (HP:0100299), Urinary bladder sphincter dysfunction (HP:0002839). | 50 | F | P | MYOT | AD | Myopathy, myofibrillar, 3 (OMIM:609200) |
| P30 | DM | Distal lower limb muscle weakness (HP:0009053), EMG: myopathic abnormalities (HP:0003458), Progressive proximal muscle weakness (HP:0009073). | 44 | M | P | MYOT | AD | Myopathy, myofibrillar, 3 (OMIM:609200) |
| P31 | UM | Generalized hypotonia (HP:0001290), Triangular mouth (HP:0000207), Long face (HP:0000276), High, narrow palate (HP:0002705), Scoliosis (HP:0002650), Joint hypermobility (HP:0001382), Hyperextensible skin (HP:0000974). | 0-1 | F | N | PLOD1 | AR | Ehlers-Danlos type 4 (OMIM:225400) |
| P32 | CMyop | Progressive proximal muscle weakness (HP:0009073), Skeletal muscle atrophy (HP:0003202), Scoliosis (HP:0002650), Hyperlordosis (HP:0003307), Areflexia of lower limbs (HP:0002522), Restrictive deficit on pulmonary function testing (HP:0002111), Hyporeflexia of upper limbs (HP:0012391), EMG: myopathic changes (HP:0003458). | 18 | M | N | RAPSN | AR | Myasthenic syndrome, congenital, 11, associated with acetylcholine receptor deficiency (OMIM: 616326) |
| P33 | UM | Intellectual disability, mild (HP:0001256), Specific learning disability (HP:0001328), Intellectual disability, moderate (HP:0002342), Delayed gross motor development, (HP:0002194), Congenital contracture (HP:0002803), Short stature (HP:0004322), Proximal amyotrophy (HP:0007126). | 0-1 | M | N | RYR1 | AD | Congenital autosomal dominant myopathy (OMIM:180901) |
| P34 | CMyop | Delayed gross motor development (HP:0002194), Scoliosis (HP:0002650), Progressive distal muscle weakness (HP:0009063), Proximal muscle weakness (HP:0003701), Pelvic girdle muscle weakness (HP:0003749). | 0-1 | F | N | RYR1 | AR | Central core disease (OMIM:117000) |
| P35 | CMyop | Muscle weakness (HP:0001324), EMG abnormality (HP:0003457), Muscular hypotonia (HP:0001252). | 0-1 | M | N | RYR1 | AR | Central core disease (OMIM:117000), Minicore myopathy with external ophthalmoplegia (OMIM:255320), congenital neuromuscular disease with uniform type 1 fiber (OMIM:117000),  Congenital myopathies with central nuclei (PMID: 20839240). |
| P36 | CMyot | Muscle cramps (HP:0003394), EMG abnormality (HP:0003457), Myotonia with warm-up phenomenon (HP:0003740). | 17 | F | P | SCN4A | AD | Myotonia congenita, atypical, acetazolamide-responsive (OMIM:608390), Paramyotonia congenital (OMIM:168300). |
| P37 | LGMD | Muscular dystrophy (HP:0003560),  Proximal muscle weakness, EMG: myopathic changes (HP:0003458). | 10 | F | N | SGCA | AR | Muscular dystrophy, limb-girdle, autosomal recessive 3 (OMIM:608099) |
| P38 | CMyop | Muscular weakness (HP:0001324), Neonatal respiratory distress (HP:0002643), Congenital myopathy,  EMG: myopathic changes (HP:0003458). | 3 | F | N | SYT2 | AD | Myasthenic syndrome, congenital, 7, presynaptic (OMIM:616040) |
| P39 | UM | EMG abnormality (HP:0003457), Gait disturbance (HP:0001288), Proximal muscle weakness in lower limbs (HP:0008994). | 48 | M | N | TRIM32 | AR | Muscular dystrophy, limb-girdle, autosomal recessive 8 (OMIM:254110) |
| P40^R^ | LGMD | Waddling gait (HP:0002515), Muscular dystrophy (HP:0003560), Limb-girdle muscular dystrophy (HP:0006785), Proximal lower limb amyotrophy (HP:0008956), Decreased patellar reflex (HP:0011808). | 23 | F | P | TTN | AR | Muscular dystrophy, limb-girdle, autosomal recessive 10 (OMIM:608807) |
| P41^R^ | LGMD | Limb-girdle muscle atrophy (HP:0003797), Limb-girdle muscle weakness (HP:0003325), Waddling gait (HP:0002515). | 15 | M | P | TTN | AR | Muscular dystrophy, limb-girdle, autosomal recessive 10 (OMIM:608807) |
| P42 | UM | Cleft palate (HP:0000175), Pectus excavatum (HP:0000767, Generalised hypotonia (HP:0001290), Toe clinodactyly (HP:0001863), Thoracolumbar scoliosis (HP:0002944), EMG: myopathic abnormalities (HP:003458), Short stature (HP:0004322), Tricuspid regurgitation (HP:0005180), Contractures of the large joints (HP:0005781), Absent phalangeal crease (HP:0006109), Severe muscular hypotonia (HP:0006829). | 0-1 | F | N | TTN | AR | Muscular dystrophy, limb-girdle, autosomal recessive 10 (OMIM:608807), Salih myopathy (OMIM:611705) |
| P43 | UM | Limb-girdle muscle weakness (HP:0003325), Waddling gait (HP:0002515), Lower limb muscle weakness (HP:0007340), Rimmed vacuoles (HP:0003805), Limb-girdle muscular dystrophy (HP:0006785), Myopathy (HP:0003198). | 27 | M | N | TTN | AR | Muscular dystrophy, limb-girdle, autosomal recessive 10 (OMIM:608807), Salih myopathy (OMIM:611705) |

LGMD - limb girdle muscular dystrophy; CMyop - congenital myopathy; DMD – Duchenne /Becker muscular dystrophy; CMyot - congenital myotonia; MitM – mitochondrial myopathy; DM - distal myopathy; UM - unspecified myopathy; N – negative, P – positive, M – male, F – female); XR: X-linked recessive; AD: autosomal dominant; AR: autosomal recessive, Mt - mitochondrial
